# The Brachyury Gly177Asp SNP Is not Associated with a Risk of Skull Base Chordoma in the Chinese Population

**DOI:** 10.3390/ijms141121258

**Published:** 2013-10-25

**Authors:** Zhen Wu, Ke Wang, Liang Wang, Jie Feng, Shuyu Hao, Kaibing Tian, Liwei Zhang, Guijun Jia, Hong Wan, Junting Zhang

**Affiliations:** 1 Skull Base and Brainstem Tumor Division, Department of Neurosurgery, Beijing Tian Tan Hospital, Capital Medical University, Tiantan Xili 6, Beijing 100050, China; E-Mails: wuzhen1966@aliyun.com (Z.W.); wangke15903@163.com (K.W.); saintage7@126.com (L.W.); shuyuhao@aliyun.com (S.H.); tiankaibing@126.com (K.T.); zlwtt@hotmail.com (L.Z.); jgjttyy@aliyun.com (G.J.); 2 Beijing Neurosurgery Institute, Capital Medical University, Tiantan Xili 6, Beijing 100050, China; E-Mails: fj79330@163.com (J.F.); wanhong@163.com (H.W.)

**Keywords:** skull-base chordoma, brachyury gene, rs2305089, single-nucleotide polymorphism

## Abstract

A recent chordoma cancer genotyping study reveals that the rs2305089, a single nucleotide polymorphism (SNP) located in brachyury gene and a key gene in the development of notochord, is significantly associated with chordoma risk. The brachyury gene is believed to be one of the key genes involved in the pathogenesis of chordoma, a rare primary bone tumor originating along the spinal column or at the base of the skull. The association between the brachyury Gly177Asp single nucleotide polymorphism (SNP) and the risk of skull base chordoma in Chinese populations is currently unknown. We investigated the genotype distribution of this SNP in 65 skull-base chordoma cases and 120 healthy subjects. Comparisons of the genotype distributions and allele frequencies did not reveal any significant difference between the groups. Our data suggest that the brachyury Gly177Asp SNP is not involved in the risks of skull-base chordoma, at least in the Chinese population.

## 1. Introduction

Chordoma is a rare neoplasm that is thought to be derived from the remnants of the notochord [1]. It is a mildly-aggressive malignant tumor that often infiltrates the adjacent tissue, making it difficult to resect the tumor completely [2–4]. About 32% of chordomas occur in the skull base region, where most vital structures are involved, including cranial nerves, and vessels of the brain and brain stem. Due to the involvement of critical structures, total removal of the tumor in this region is nearly impossible, and the tumor often recurs after initial treatment over time [1].

The brachyury gene, also known as the T gene, encoding the transcriptional factor T, is located at 6q27. It is a well-conserved gene and controls the development of the notochord [5]. Normally, the T gene is only expressed in the early stages of notochord development, and is silenced in the developed nucleus pulposus [5]. Pathologic evaluations have indicated strong expression of the T gene in both sporadic and familial chordomas [5–8]. The results of *in vitro* cell line experiments, in which the expression of the T/brachyury homolog in the U-CH1 chordoma-derived cell line was silenced, resulted in inhibition of growth and the acquisition of a senescence-like phenotype [7].

A recent study reported that the brachyury Gly177Asp SNP (rs2305089 SNP) is strongly associated with chordoma in European populations [9]. The study enrolled 40 individuals with chordoma, and using whole-exome and Sanger sequencing of T exons, found that a common non-synonymous SNP, rs2305089, of the brachyury gene was a risk factor, with subjects carrying an A allele likely to have a higher risk of developing chordoma (odds ratio [OR] = 5.3; *p* = 4.6 × 10^−^^12^) [9]. The study highlighted the significant impact of T expression in the development of chordoma. However, the differences between the frequencies of the rs2305089 SNP between European and Chinese populations reported in the PubMed Database, with an almost complete reversal of the A and G allele frequencies between the two populations, made the conclusion of the study less applicable in other populations, especially in Chinese populations. Hence, in the present study, we measured the distribution of the rs2305089 SNP in the Chinese population, both in healthy subjects and in patients with skull-base chordoma, and evaluated the association of this SNP with the skull base chordomas in Chinese subjects.

## 2. Results and Discussion

### 2.1. Results

Relevant sequence data for the rs2305089 SNP was obtained for all study subjects, except for one case in the control group. Genotype and allele frequencies were in Hardy-Weinberg equilibrium in all groups, *p* > 0.05 (Table 1). Allele and genotype frequencies of the brachyury rs2305089 SNP did not differ significantly between the skull-base chordoma and control groups. The frequencies of genotypes G/G, G/A, and A/A in the control group were 40.8%, 48.3%, and 10.8%, respectively, while in the chodroma group, they were 38.5%, 49.2%, and 12.3%, respectively (*p* = 0.929) (Table 2).

Compared to the G/G genotype, the most common genotype in the study population, the A/A and G/A genotype did not influence the risk of skull-base chordoma development (95% CI, 0.488–1.680; *p* = 0.753) (Table 2). The A/A and G/A genotypes were combined in the analysis because of the low frequency of the A/A genotype. The most frequent allele, G, was also not associated with the development of skull-base chordoma (95% CI, 0.590–1.434; *p* = 0.712) (Table 2). Analysis by the gender-specific distribution in the genotypes and allele frequencies showed no correlations of either gender with specific genotypes or allele frequencies (Table 3).

The clinical and pathological characteristics are shown in Table 4. The association analyses were performed for the skull-base chordoma cases alone, by dividing the patients into two groups according to their gender, age at operation, primary or non-primary tumor at diagnosis, and histological grade. The analysis of association of the chosen clinicopathological features, using a logistic regression model, indicated no significant association between the different genotypes at rs2305089 with any of the features analyzed, including the age at operation. The G allele was likely, but failed to be associated with those aged more than 40 years (χ^2^ = 0.06; *p* = 3.530) (Table 4).

### 2.2. Discussion

The brachyury gene, a conserved gene that controls the development of the notochord, was found to be expressed in chordomas, while being silenced in notochord remnants or ecchordosis physaliphora (EP) [5]. Also, studies of familial and sporadic chordomas reported that the duplication or gain of the T locus were a common phenomenon in chordomas [5–8]. Presneau *et al*. demonstrated that the silencing of the brachyury gene in the UCH-1 cell line resulted in a decrease in cell proliferation [7], which, to date, is the most direct evidence of the effect of this gene on chordoma and its pathogenesis. The brachyury gene, acting as a master regulator of an elaborate oncogenic transcriptional network encompassing diverse signaling pathways, including components of the cell cycle and the extracellular matrix, is of great interest to researchers studying chordomas [10].

A recent study by Pillay *et al*. [9] suggested that there was a significant difference in the distribution of the rs2305089 Gly177Asp SNP in European populations and chordoma patients, with allelic ORs of 6.1 in the discovery sets and 5.3 in the total set of subjects. This was an extraordinary result that showed that the single nucleotide change in rs2305089 was strongly associated with chordoma. The possible reason, as the authors suggested, was that the A alteration was associated with different expression levels of T and its downstream targets.

However, there is a converse ratio of the G and A alleles of rs2305089 between the Chinese and European populations, as shown in Table 1. The A allele occurs at a higher frequency in European populations, but is only a minor frequency site in Chinese populations. Although it was suggested that individuals of Asian ancestry may carry the A allele at a relatively lower frequency compared to those of European ancestry [1], there is still a paucity of evidence for a positive association between the incidence of chordomas and the rs2305089 Gly177Asp SNP.

In the present case-controlled study, only patients with skull-base chordoma were enrolled, which is a different study population than that in the study by Pillay *et al*. [9], in which patients with all subtypes of chordomas were enrolled. The 65 patients with pathologically-confirmed skull-base chordomas enrolled in this study had a median age (37.9 ± 14.4 years) and gender distribution (male:female = 1.17:1) that was similar to those reported in previous reports [1–3]. The frequency of the A and G allelles were similar between the chordoma and control groups (Table 2). There was no significant association between the controls and cases in present study. As the skull-base chordoma group had a slight male predominance, a correlation analysis according to the different gender was performed. The data showed no obvious association between the developing of skull-base chordomas and the Gly177Asp SNP, regardless of the gender of the patients. Then, an association analysis was conducted between different clinicopathological parameters of the patients in the chordoma group and the Gly177Asp SNP. A moderately-positive relationship was noticed between the age at the time of the surgery; however, the age at the time of operation was not related to or was further along compared to the age of diagnosis or the time of onset of the symptoms. Therefore, further investigation is needed to clarify the correlation between the age of the patient, at the time of diagnosis/onset of symptoms/surgery, and the Gly177Asp SNP in the brachyury gene.

The present case-control study was performed with limited cases due to the rarity of the incidence of the skull base chordomas, which may resulted to the false negative findings, as the statistic power of the present study was not enough. However, due to the similar risk allele frequency of the allele A between the case group and the control group in the Chinese population, there lies a statistic dilemma, especially on the condition that rarity of the skull base chordoma [1]. The conclusion that the negative association between the brachyury gene rs2305089 and the skull base chordoma should be considered under caution.

The results discussed above do not indicate any correlation of the brachyury rs2305089 SNP with the risk of skull-base chordoma in Chinese patients with limited cases. However, linkage disequilibrium was not included in this study for only one site of SNP was examined, and the functional study was not performed because of the negative results. Taken together, the data suggest that the site of rs2305089 Gly177Asp might not be a functional SNP for the skull-base chordoma, at least in Chinese patients. It was therefore suggested that, firstly, the risk factor of the skull base chordoma may be different from the other parts of chordoma, secondly, if the brachyury is indeed associated with skull base chordoma, there may other SNP sites, which may be in linkage disequilibrium, plays the role of functional SNP in skull base chordoma. Another suggestion is that, if the brachyury gene and Brachyury protein were indeed critical components that are altered during the development of chordoma, there may be other mechanisms through which they may be involved, rather than an association through the SNP alone, as has been suggested by Le *et al*. [11].

While many researchers had found the positive expression of brachyury gene in chordoma [5,6], the mechanism of this gene and its function in chordoma is still under debate. Using a variety of analyses, including fluorescence in site hybridization (FISH), quantitative PCR (qPCR) and array CGH (aCGH), Presneau *et al*. [7] analyzed 181 chordoma specimens, their concluded that chromosomal aberrations resulting in gain of the T locus are common in sporadic chordomas. The familiar chordoma research of Yang *et al*. [8] identified T gene as a major susceptibility gene using combined genetic linkage and high-resolution array-CGH analyses. However, they found no disease-associated mutations in this gene. Also, their results showed that three familiar chordoma patients did not have brachyury gene duplications. This result was further supported by Le *et al*. [11], who reported the copy number analysis of 21 sporadic chordomas with the results of most sporadic tumors not associated with brachyury duplication or amplification. It seems that the brachyury gene maybe a target gene in the pathogenesis of the chordoma, which resulted the positive expression of brachyury compared to the remnant of the notochord tissue [5].

In addition, the brachyury gene was expressed in a number of tumors derived from the mesoderm, such as colorectal cancer [12] and hemangioblastoma [13]. Recent studies revealed that brachyury is a prognostic factor for some cancers rather than just a definitive diagnostic marker of chordoma [12,13]. For instance, brachyury expression was related to the epithelial-to-mesenchymal transition (EMT), which is thought to be vital for metastasis [14]. If the brachyury protein is the “break point”, as it was once supposed [5], further investigation of cellular pathways, including the wnt signaling, β-catenin/transcription factor 4 (TCF4) complex-driven, and TGF-β pathways, are needed [14–16]. Meanwhile, a comparison of the tumors which express the brachyury gene might give a clue to better understand the chordoma.

In summary, the present study did not support that the rs2305089 SNP was a risk factor for skull base chordoma in Chinese population, with limited statistic power. Further studies on the function of the brachyury gene are therefore highlighted, especially those using chordoma cell lines and familiar chordomas.

## 3. Experimental Section

### 3.1. Study Population

This study was a population-based case-controlled study performed in Beijing, China. From July 2010 to September 2012, 65 pathologically-confirmed skull-base chordoma patients treated at the University Hospital of the Tian Tan hospital, Capital Medical University, were enrolled in this study. Among the patients, the male: female ratio was 1.17:1 (35:30), the age ranged from 8 to 69 years (median, 37.9 ± 14.4 years). In the control group, 121 healthy individuals were enrolled, with a male: female ratio of 0.90:1 (57:63) and of ages ranging from 25 to 79 years (median, 45.9 ± 9.4 years). Blood samples (5 mL) were collected from each of the patients and healthy individuals. All patients provided informed written consent for participation in the study.

### 3.2. DNA Extraction and Genotyping

DNA extraction was performed using proteinase K and phenol-chloroform, using the TIANamp Blood DNA Kit (DP318; TIANGEN Biotech (Beijing) Co., Ltd., Beijing, China). DNA samples were stored at the 4 °C. Primers were designed by Sangon Biotech (Shanghai, China) Co., Ltd. The sequence containing the polymorphic site was amplified by PCR using the following primers: forward, 5′-CTC CTC CAG AAT CTC CAC GG-3′ and reverse, 5′-ACC AAT CCT GTA TCT GTC TCC CT-3′. Genotyping was performed by Sangon Biotech (Shanghai, China) Co., Ltd., with the method of sequencing.

### 3.3. Statistical Analysis

The independence of alleles (Hardy-Weinberg equilibrium) was ensured using the χ^2^ test. The genotype distribution and the allele frequencies between patients and control subjects were compared using the χ^2^ and Fisher’s exact tests. The OR and 95% confidence intervals (CIs) were calculated with the SPSS statistical software (SPSS Version 13.0 software, SPSS Inc., Chicago, IL, USA).

## 4. Conclusion

In summary, the results of the present study do not support the hypothesis that the A or G allele alone was associated with the skull base chordoma, at least in the Chinese population. Additional studies need to be conducted to further evaluate our findings.

## Figures and Tables

**Table 1:** The Hardy-Weinberg equilibrium of rs2305089 in different nations.

Rs2305089	G allele	A allele	χ^2^	*p*-Value
HCB *	0.640	0.360	-	-
CEU *	0.410	0.590	-	-
YRI *	0.797	0.203	-	-
Chordoma group	0.63	0.37	0.21	>0.05
Control group	0.65	0.35	0.47	>0.05

HCB, Asian; CEU-European; YRI, Sub-Sahara African;

* , The results were obtained from the PubMed database using the search terms “SNP rs2305089”.

**Table 2:** Genotype and allele frequencies of the brachyury Gly177Asp single-nucleotide polymorphism in the skull-base chordoma and control groups.

Genotype	Cases (*n* (%))	Controls (*n* (%))	*p*-Value	95% CI
G/G	25 (38.5)	49 (40.8)	0.929	
G/A	32 (49.2)	58 (48.3)		
A/A	8 (12.3)	13 (10.8)		
GA + AA *	40 (61.5)	71 (59.1)	0.753	0.488–1.680
G177 Freq	0.63	0.65	0.712	0.590–1.434

Freq, allele frequency; CI, confidence interval;

* , Statistic power: 88%, under the supposed OR value (OR = 3).

**Table 3:** The genotype and allele frequencies of the brachyury Gly177Asp single-nucleotide polymorphism in the skull-base chordoma and control groups, sorted based on the gender of the subject/patient.

Gender	Genotype	Cases (*n* (%))	Controls (*n* (%))	*p*-Value	95% CI
Male	G/G	14 (40.0)	28 (49.1)	0.937	
	G/A	16 (45.7)	22 (38.6)		
	A/A	5 (14.3)	7 (12.3)		
	G177 Freq	0.63	0.68	0.438	0.418–1.460

Female	G/G	11 (36.7)	21 (33.3)	0.940	
	G/A	16 (53.3)	36 (57.1)		
	A/A	3 (10.0)	6 (9.5)		
	G177 Freq	0.63	0.62	0.851	0.563–2.009

Freq, allele frequency; CI, confidence interval.

**Table 4:** Association of the rs2305089 genotype and clinical characteristics in skull-base chordoma.

	Total	Genotype			Allele count		Frequency		*p*-Value

		GG	GA	AA	G	A	G	A	
Gender									
Male	35	14	16	5	44	26	0.63	0.37	0.955
Female	30	11	16	3	38	22	0.63	0.37	
Age at the time of the operation (years)									
≤40	35	10	19	6	39	31	0.56	0.44	0.060
>40	30	15	13	2	43	17	0.72	0.18	
Outcome									
Primary	48	18	27	3	63	33	0.66	0.34	0.312
Recurrence	17	7	5	5	19	15	0.56	0.44	
Histology									
Conventional	62	25	30	7	80	44	0.65	0.35	/
Chondroid	2	0	2	0	2	2	0.5	0.5	
De-differential	1	0	0	1	0	2	0	1.0	

“/”: the chi-square test was not performed due to the rare number of the cases.
